# Identification of BAHD‐acyltransferase enzymes involved in ingenane diterpenoid biosynthesis

**DOI:** 10.1111/nph.70388

**Published:** 2025-07-17

**Authors:** Carsten Schotte, Matilde Florean, Tomasz Czechowski, Alison Gilday, Ryan M. Alam, Kerstin Ploss, Jens Wurlitzer, Yi Li, Prashant Sonawane, Ian A. Graham, Sarah E. O'Connor

**Affiliations:** ^1^ Department of Natural Product Biosynthesis Max Planck Institute for Chemical Ecology 07745 Jena Germany; ^2^ Department of Biology, Centre for Novel Agricultural Products University of York Heslington York YO10 5DD UK; ^3^ Department of Biochemistry, College of Agriculture, Food and Natural Resources University of Missouri Columbia MO 65211 USA

**Keywords:** BAHD‐acyltransferase, diterpene, *Euphorbia peplus*, ingenol‐3‐angelate, medicinal plant, natural product biosynthesis

## Abstract

- The plant family Euphorbiaceae is an abundant source of structurally complex diterpenoids, many of which have reported anticancer, anti‐HIV, and anti‐inflammatory activities. Among these, ingenol‐3‐angelate (**1a**; tradename: Picato®), isolated from *Euphorbia peplus*, has potent antitumor activity.
- We report the discovery and characterization of the first genes linked to committed steps of ingenol‐3‐angelate (**1a**) biosynthesis in *E. peplus*. Using pathway reconstitution in *Nicotiana benthamiana* and *in vitro assays* with recombinant enzymes, we identified two genes whose products catalyze the addition of angelyl‐CoA (**9a**) to the ingenol (**5**) scaffold, producing ingenol‐3‐angelate (**1a**). We also identified three genes whose products catalyze acetylation of ingenol‐3‐angelate (**1a**) to ingenol‐3‐angelate‐20‐acetate (**2**). Virus induced gene silencing (VIGS) suggests considerable functional redundancy in the *E. peplus* genome for this enzymatic step. We also identified three genes whose products can catalyze acetylation of ingenol‐3‐angelate (**1a**) to ingenol‐3‐angelate‐20‐acetate (**2**). In this case, virus‐induced gene silencing (VIGS) indicates considerable functional redundancy in the *E. peplus* genome of genes encoding this enzymatic step.
- We demonstrate using VIGS that just one of these genes, *EpBAHD‐08*, is essential for this angeloylation in *E. peplus*. VIGS of the second gene, *EpBAHD‐06*, has a significant effect on jatrophanes rather than ingenanes in *E. peplus*.
- This work paves the way for increasing ingenol‐3‐angelate (**1a**) levels *in planta* and provides a foundation for the discovery of the remaining genes in the biosynthetic pathway of these important molecules.

## Introduction

The Euphorbiaceae are one of the largest families of plants with more than 7500 species reported to date (Zhan *et al*., 2022; Johnson *et al*., 2023). Notably, the majority of Euphorbiaceae plants produce a milky latex that is rich in biologically active diterpenoids (Johnson *et al*., 2023). From the genus *Euphorbia*, > 1500 diterpenoids with more than 30 different skeletal backbones have been isolated (Vasas & Hohmann, 2014; Xu *et al*., 2021; Zhan *et al*., 2022). These skeletal backbones have exceptional structural complexity, and members of this natural product family range in the number of ring systems, degree of oxygenation, stereochemistry, and esterification pattern (Luo *et al*., 2016).

**Fig. 1:**
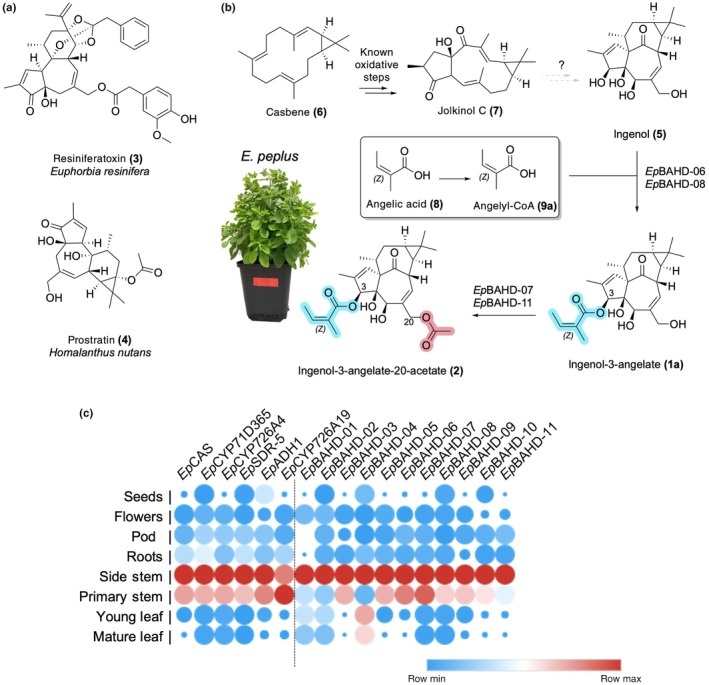
Diterpene biosynthesis in the *Euphorbia* genus. (a) Clinically important diterpenoids isolated from various *Euphorbia* species. (b) Known, proposed, and newly discovered steps in *Euphorbia peplus* diterpene biosynthesis. Genes identified in this study catalyze formation of ingenol‐3‐angelate (**1a**) from ingenol (**5**) (*Ep*BAHD‐06 and *Ep*BAHD‐08); and ingenol‐3‐angelate‐20‐acetate (**2**) from ingenol‐3‐angelate (**1a**) (*Ep*BAHD‐07 and *Ep*BAHD‐11). (c) Expression profiles of genes involved in jolkinol C (**7**) biosynthesis and BAHD‐acyltransferases identified in the course of this study. Red corresponds to maximum expression, and blue corresponds to minimum expression levels per gene across different tissues (expression measured in fragments per kilobase of exon per million of mapped fragments). The heatmap was created with Morpheus (https://software.broadinstitute.org/morpheus).

Clinically important diterpenoids from the *Euphorbia* genus include resiniferatoxin (**3**), a transient receptor vanilloid 1 agonist, that is currently being investigated for treatment of an overactive bladder and chronic pain (phase III clinical trial), and prostratin (**4**), which may have applications in clearing latent virus reservoirs in HIV infections (Fig. 1a) (Johnson *et al*., 2008; Brown, 2016; Zhan *et al*., 2022). Ingenol‐3‐angelate (**1a**) is perhaps the most well‐known diterpene produced by *Euphorbia* (*Euphorbia peplus*; Fig. 1b). This compound was approved in 2012 by the Food and Drug Administration (FDA) for the treatment of the precancerous skin condition actinic keratosis (Picato®) but was discontinued in 2020 in the course of a phase IV clinical trial when it appeared that this compound increased the incidence of skin cancer (Alves *et al*., 2022). However, ingenol‐3‐angelate (**1a**) is still being explored in the treatment of HIV infections (Jiang *et al*., 2015). Additionally, members of this type of diterpenoid (ingenane class) appear to be a rich source of biologically active compounds (Alves *et al*., 2022). Therefore, ingenol‐3‐angelate (**1a**) and related compounds continue to be of high interest for pharmaceutical development.

*Euphorbia*‐derived diterpenoids typically accumulate to low levels *in planta*: Ingenol‐3‐angelate (**1a**) is present at 1.1 mg per kg fresh weight in aerial tissues (Hohmann *et al*., 2000). Chemical syntheses of these structurally complex diterpenes have been reported, but these methods suffer from low yields and long linear reaction sequences (> 10 steps) (Jørgensen *et al*., 2013; Vasilev *et al*., 2022). While semisynthetic approaches toward **1a** from more readily available plant intermediates have been reported (e.g. three steps from ingenol (**5**); Supporting Information Fig. S1), these approaches still depend on expensive and nonabundant starting materials (Liang *et al*., 2012).

Therefore, new methods are needed to produce complex diterpenoids at scale. The use of metabolic engineering methods to produce these compounds in microbial hosts could potentially meet this need. Efforts to engineer *Euphorbia* diterpenoid production, however, are limited since the biosynthesis of these terpenes is not well‐understood, and most biosynthetic genes that are responsible for the production of these compounds have not been identified.

It is believed that all *Euphorbia*‐specific diterpenoids derive from the bicyclic diterpene casbene (**6**) (Fig. 1B), which is the initial product of the class I diterpene synthase, casbene synthase (*Ep*CAS) (Kirby *et al*., 2010). The casbene‐derived *Euphorbia* diterpenoids are classified by increasing cyclization of the diterpene backbone, giving rise to jatrophane, lathyrane, tigliane, daphnane, and ingenane classes, with the latter being the most complex diterpenoids (Luo *et al*., 2016; Zhan *et al*., 2022) (Fig. S2). Only the biosynthetic genes that convert casbene (**6**) to the simple lathyrane‐type diterpenoids jolkinol C (**7**) (Figs 1b, S3) and jolkinol E (**S3**) have recently been identified (Fig. S3) (King *et al*., 2014, 2016; Luo *et al*., 2016; Wong *et al*., 2018). Silencing of some of these genes in *E. peplus* using virus‐induced gene silencing (VIGS) confirmed the role of casbene as a precursor and suggested that jolkinol C (**7**) may be an on‐pathway intermediate for ingenol‐3‐angelate (**1a**), but the majority of downstream acting biosynthetic genes in this pathway and related diterpenoid pathways are unknown (Czechowski *et al*., 2022). Notably, the production of jolkinol C (**7**) in baker's yeast at titers of 800 μg ml^−1^ has been reported, suggesting that production by heterologous reconstitution for this class of diterpenes is feasible (Wong *et al*., 2018), if subsequent biosynthetic steps are elucidated.

Here, we identify five acyltransferases that derivatize the ingenol (**5**) scaffold to form both ingenol‐3‐angelate (**1a**) and ingenol‐3‐angelate‐20‐acetate (**2**) (Fig. 1b), the last predicted enzymatic steps in this biosynthetic network. Transient gene expression in the heterologous host plant *Nicotiana benthamiana* and *in vitro* enzyme assays confirms catalytic activity toward ingenane production. Notably, with the exception of two acyltransferases from *Euphorbia lathyris* (Zhao *et al*., 2025), no acyltransferases had previously been identified in any *Euphorbia* diterpene pathways despite the fact that acylation is one of the most predominant decorations of the *Euphorbia* diterpenoids (Fig. S4). This discovery fills a gap in the biosynthesis of this important class of compounds and, furthermore, sets the stage for further mining of omics data to identify the remaining missing genes involved in ingenol‐3‐angelate (**1a**) biosynthesis.

## Materials and Methods

Comprehensive descriptions of materials and methods employed in all experiments are included in Methods S1.

### Plant material and growth

*E. peplus* L. plants were grown in climate chambers (12 h : 12 h, light : dark photoperiod). Plants were kept from 12 to 7 pm at 24°C (50–55% relative humidity) and during the night at 22°C (60% relative humidity). Eight‐week‐old plants were used for transcriptomic and metabolomic studies. *N. benthamiana* plants were cultivated as recorded previously (Schotte *et al*., 2023). Briefly, plants were maintained at 22°C with 60% relative humidity under a 16 h : 8 h, light : dark photoperiod. Tobacco plants were used for infiltration at 3–4 wk post germination.

### *De novo* transcriptome assembly and gene candidate identification

Total RNA was isolated from bulk plant tissues using commercially available kits and procedures. Three biological replicates of each tissue were used. Standard mRNA library preparation, Illumina 2 × 150 bp sequencing, and *de novo* transcriptome assembly as well as functional annotation were performed by *BGI Genomics*. Acyltransferase genes were identified by co‐expression with the known gene casbene synthase (GenBank: KJ026362.1; Pearson's correlation coefficient *r* ≥ 0.75).

### *Agrobacterium tumefaciens*‐mediated transient transformation of *N. benthamiana*

Transient expression of acyltransferase gene candidates in tobacco leaves was performed as recently reported (Schotte *et al*., 2023). Briefly, *Agrobacterium tumefaciens* GV3101 strains carrying the construct of interest were grown overnight in Lysogeny Broth (LB) medium supplemented with appropriate antibiotics. Bacterial cells were collected by centrifugation and washed twice with infiltration buffer containing 100 μM of acetosyringone. The cells were then resuspended in the same buffer and adjusted to an OD_600_ = 0.3 for single‐strain infiltrations, or to < 1.0 for mixed‐strain infiltrations. Following a 60‐min incubation in the dark, the suspension was infiltrated into the abaxial side of tobacco leaves using a needleless syringe. Three days later, 100–300 μM of substrate was infiltrated into the same leaves. After an additional 2 d, leaves were harvested and extracts analyzed by liquid chromatography‐mass spectrometry. Each candidate was tested at least two times in two biological replicates.

### Heterologous expression of candidate genes in *E. coli* and *in vitro* assays

Genes with acyltransferase activity were recombinantly produced in *Escherichia coli* as previously reported (Schotte *et al*., 2023). Expression plasmids carrying the gene of interest were transformed into *E. coli* BL21(DE3) cells. Single colonies were grown overnight, and an aliquot of the seed culture was used to inoculate 100–1000 ml of 2xYT‐medium. Cultures were incubated at 37°C and 200 rpm until reaching an OD_600_ of 0.4–0.6. Protein expression was induced by adding isopropyl‐*β*‐d‐1‐thiogalactopyranoside to a final concentration of 200 μM, followed by cultivation at 18°C for up to 24 h. Recombinant acyltransferase enzymes were purified using standard Ni‐NTA affinity chromatography protocols. *In vitro* assays contained 2 μg of recombinant protein, 200 μM of the respective CoA donor, and 100 μM of ingenol, ingenol‐3‐angelate, or ingenol‐3‐angelate‐2‐acetate in 25 mM phosphate buffer (pH 7.5). After incubation, the assays were stopped by the addition of MeOH, and filtered solutions were analyzed by liquid chromatography‐mass spectrometry.

### Virus‐induced gene silencing of acyltransferase genes

VIGS was performed as recently reported (Czechowski *et al*., 2022). The Chlorota 42 marker gene (*EpCH42*) was used as a control. Briefly, < 200 bp from nonconserved regions of the targeted acyltransferase genes were cloned into the pTRV2‐*EpCH42*‐VIGS construct using In‐Fusion cloning. Constructs were verified by Sanger sequencing and transformed into *A. tumefaciens* GV3101. Strains carrying either pTRV1, pTRV2‐*EpCH42*‐VIGS, or one of five target‐gene constructs were grown overnight and used to inoculate 50 ml of LB medium, followed by overnight culture at 28°C and 220 rpm. These cells were harvested by centrifugation and resuspended in infiltration buffer containing 200 μM of acetosyringone to a final OD_600_ of 2.5, then incubated for 2 h at 28°C and 110 rpm. Equal volumes of each pTRV2‐derived strain were mixed with the pTRV1 culture and infiltrated into both cotyledons of 9‐d‐old *E. peplus* seedlings. Plants were maintained under a 16 h : 8 h, 25°C : 22°C, light : dark cycle (day : night). Six weeks post infiltration, chlorotic parts of leaf and stem tissues were collected separately. Fresh plant material was pooled from six to eight independent plants to form one biological replicate, flash‐frozen in liquid N_2_, and stored in −80°C.

## Results and Discussion

*Euphorbia peplus* was cultivated in climate chambers until mature seeds ripened. Eight weeks after initiating cultivation, identical tissue samples were collected for both metabolomics and transcriptomics (mature leaves, young leaves, primary stem, side stem, pods, flowers, roots, seeds (mature seeds were collected later after ripening), and latex). Metabolomics analysis revealed the presence of ingenol‐3‐angelate (**1a**) in all tested tissues, but **1a** was predominantly found in the latex (Fig. S5). Note that no RNA could be isolated from latex over the course of this study, preventing transcriptomic analysis of this material. A similar analysis for **2** indicated that this metabolite was also predominantly located in the latex, but again, traces of **2** were found in all tissues (Fig. S6).

### Gene candidate identification and expression in *Nicotiana benthamiana*

Previous work in *E. peplus* revealed that two lathyranes, jolkinol C **(7)** and jolkinol E, are potential intermediates in the pathways to the ingenane and jatrophane classes of diterpenoids, respectively (Czechowski *et al*., 2022). An analysis of bulk tissue transcriptomic data revealed that the previously identified jolkinol C and E biosynthetic genes (Fig. S3) are primarily expressed in stem tissue (Fig. 1c), which is enriched in ingenanes and jatrophanes. Based on these observations, we selected candidate BAHD‐acyltransferase genes for functional characterization based on their co‐expression with casbene synthase, which catalyzes the first committed step in the biosynthesis of both jolkinol C and jolkinol E (Fig. S3; Table S1).

**Fig. 2:**
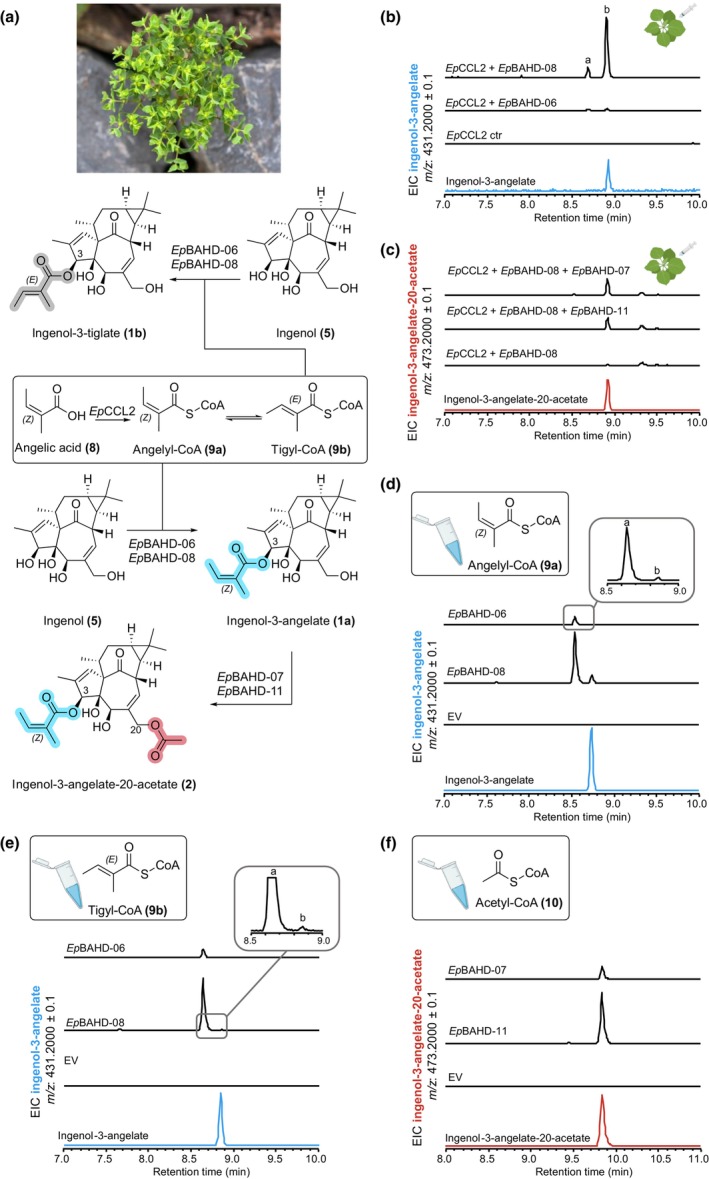
Enzyme activity assays of acyltransferases from *Euphorbia peplus*. (a) Enzymatic reactions observed in this study. (b) Tobacco infiltration of a dedicated angelyl‐CoA ligase (*Ep*CCL2) (Callari *et al*., 2018), angelic acid (**8**), ingenol (**5**), and *Ep*BAHD‐06 and *Ep*BAHD‐08. Both enzymes catalyze formation of ingenol‐3‐angelate (**1a**) *in planta* (peak B). (c) Tobacco infiltration of a dedicated angelyl‐CoA ligase (*Ep*CCL2), angelic acid (**8**), ingenol (**5**), *Ep*BAHD‐08 (together affording ingenol‐3‐angelate [**1a**]), and *Ep*BAHD‐07 and *Ep*BAHD‐11. Both *Ep*BAHD‐07 and *Ep*BAHD‐11 catalyze formation of ingenol‐3‐angelate‐20‐acetate (**2**). Note that chromatography methods in panel (b) and (c) are different and that **1a** and **2** can be differentiated. (d) *In vitro* assays with *Ep*BAHD‐06 and *Ep*BAHD‐08 using angelyl‐CoA (**9a**) and ingenol (**5**) as substrates lead to formation of ingenol‐3‐angelate (**1a**) as a minor product (peak B), with the isomer ingenol‐3‐tiglate (**1b**) (peak A) as the major product. (e) *In vitro* assays with *Ep*BAHD‐06 and *Ep*BAHD‐08 using tiglyl‐CoA (**9b**) and ingenol (**5**) as substrates lead to formation of ingenol‐3‐tiglate (**1b**) (peak A). (f) *In vitro* assays with *Ep*BAHD‐07 and *Ep*BAHD‐11 using ingenol‐3‐angelate (**1a**) and acetyl‐CoA (**10**) as substrates lead to formation of ingenol‐3‐angelate‐20‐acetate (**2**). Plant art and depiction of microcentrifuge tube in Scheme 2 (b–f) are from biorender.com (https://BioRender.com/1qhnqih).

A total of 11 BAHD‐acyltransferase candidate genes (Tables S1–S3) were selected for functional characterization. Each gene was transiently expressed in *N. benthamiana* along with an angelyl‐CoA ligase gene that had been previously shown to catalyze the formation of angelyl‐CoA (**9a**) in yeast (*Ep*CCL2) (Callari *et al*., 2018). After the transfection of the acyltransferase candidate and angelyl‐CoA ligase genes in *N. benthamiana*, the substrates ingenol (**5**) and angelic acid (**8**) were infiltrated into the leaf. Leaf tissue was then analyzed by LC‐MS to assess the conversion of ingenol (**5**) to ingenol‐3‐angelate (**1a**). From this assay, we showed that two genes, *Ep*BAHD‐06 and *Ep*BAHD‐08, catalyze the acylation of ingenol (**5**) to form ingenol‐3‐angelate (**1a**) based on comparison with an authentic standard (Fig. 2a,b). Intriguingly, both enzymes produced a minor second product that showed an identical mass and fragmentation pattern as ingenol‐3‐angelate (**1a**) (Fig. S7).

Since angelic acid (**8**) contains an alkene with *Z* configuration, we reasoned that the alkene might readily isomerize to the more stable *E* isomer, tiglic acid (Fig. 2a). The *E* to *Z* isomerization of angelic acid (**8**) has already been reported during the synthetic angeloylation of ingenol (**5**) (Liang *et al*., 2012). Therefore, we reasoned that the minor product is ingenol‐3‐tiglate (**1b**) (Fig. 2a).

We next assessed whether the angelyl‐CoA ligase gene (*Ep*CCL2) is required for the production of ingenol‐3‐angelate (**1a**) in *N. benthamiana*. Interestingly, the omission of the angelyl‐CoA ligase had no significant impact on ingenol‐3‐angelate (**1a**) formation, suggesting that *N. benthamiana* can convert angelic acid (**8**) to angelyl‐CoA (**9a**) using an endogenous enzyme (Fig. S8). Notably, the addition of angelic acid was absolutely required for the formation of **1a**, suggesting that *N. benthamiana* does not have the required pools of this acyl donor (Fig. S9).

To screen for downstream acyltransferase activity, *Ep*BAHD‐08 was again expressed in *N. benthamiana*, but this time together with the other acyltransferase candidates. This assay revealed that in combination with ingenol (**5**) and *Ep*BAHD‐08, both *Ep*BAHD‐07 and *Ep*BAHD‐11 catalyze the formation of ingenol‐3‐angelate‐20‐acetate (**2**), another well‐known, biologically active diterpenoid derivative from *E. peplus* (Alves *et al*., 2022) (Fig. 2c).

### *Escherichia coli* expression and *in vitro* assays

*In vitro* enzyme assays using purified recombinant proteins were performed to validate the catalytic activity observed after expression of these genes in *N. benthamiana* leaves. All four BAHD‐acyltransferases (*Ep*BAHD‐06, *Ep*BAHD‐07, *Ep*BAHD‐08, and *Ep*BAHD‐11) were recombinantly produced in *E. coli*. Angelyl‐CoA (**9a**) and tiglyl‐CoA (**9b**) were chemically synthesized.

In contrast to assays performed *in planta*, in these *in vitro* assays, incubation of ingenol (**5**) with angelyl‐CoA (**9a**) and *Ep*BAHD‐06 or *Ep*BAHD‐08 led to the formation of ingenol‐3‐angelate (**1a**) as a minor product (Fig. 2d). The major product was the previously observed isomer of ingenol‐3‐angelate (**1a**), which we had tentatively assumed to be ingenol‐3‐tiglate (**1b**). To confirm this, tiglyl‐CoA (**9b**) was also chemically synthesized and then incubated with ingenol (**5**) and *Ep*BAHD‐06 or *Ep*BAHD‐08. The resulting product was identical to the major product observed in the assay performed with angelyl‐CoA (**9a**) (Fig. 2e). These *in vitro* assays therefore suggest that the isomerization of the alkene moiety to the more stable *E* isomer likely occurs on angelyl‐CoA (**9a**) before the transfer of the acyl group to ingenol (**5**).

Finally, incubation of ingenol‐3‐angelate (**1a**) with acetyl‐CoA (**10**) and *Ep*BAHD‐07 or *Ep*BAHD‐11 led to the formation of ingenol‐3‐angelate‐20‐acetate (**2**) (Fig. 2f).

**Fig. 3:**
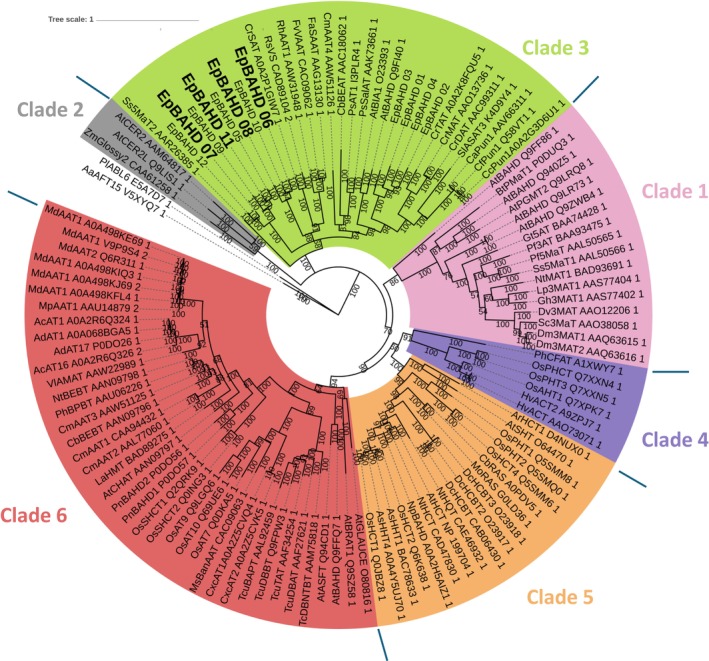
Phylogenetic analysis of acyltransferases identified from *Euphorbia peplus* in this study. Sequence alignment of amino acid sequences was performed using Muscle v.3.8.425 (Edgar, 2022). The displayed tree was then constructed with Bayesian analyses using MrBayes v.3.2.7a (Ronquist *et al*., 2012). Posterior probabilities were reported as supporting values for nodes in the trees, and the scale bar represents substitutions per site (bar indicates a branch length of 0.2.). Lines indicate the edges of the clades.

Next, we performed a phylogenetic analysis of the 11 gene candidates. A multiple sequence alignment was made with 104 functionally annotated acyltransferases from other plants and using two fungal acyltransferase genes as an outgroup (Figs 3, S10). Plant BAHD‐acyltransferases group into six distinct clades (D'Auria, 2006; Moghe *et al*., 2023) and the tested acyltransferases from *Euphorbia* form three distinct subgroups within Clade 3, a clade generally known to be involved in plant specialized metabolism (Moghe *et al*., 2023). Notably, *Ep*BAHD‐06/*Ep*BAHD‐08 and *Ep*BAHD‐07/*Ep*BAHD‐11, respectively, group in different subclades. Future studies should systematically investigate the functional activity of acyltransferases grouping in other *Euphorbia*‐specific subclades to unveil additional acyltransferases involved in the diverse acyltransferase chemistry observed in *Euphorbia* diterpenoids.

### *In planta* confirmation of diterpene biosynthetic activities of *E. peplus BAHD* genes using virus‐induced gene silencing (VIGS)

To further corroborate the function of these identified acyltransferases, VIGS (as previously established for *E. peplus* (Czechowski *et al*., 2022)) was used to silence expression of the four BAHD genes shown to have activity on ingenol (**5**) or ingenol‐3‐angelate (**1a**). To avoid off‐site targeting, low homology regions of *EpBAHD‐06, EpBAHD‐07, EpBAHD‐08*, and *EpBAHD‐11* were selected (Fig. S11) and cloned into a pTRV2 vector containing the previously described silencing marker *EpCH42* (Czechowski *et al*., 2022). *A. tumefaciens‐mediated* infiltration was then carried out in three batches: (1) targeting *EpCH42:EpBAHD‐06* and *EpCH42:EpBAHD‐08*; (2) targeting *EpCH42:EpBAHD‐07* and *EpCH42:EpBAHD‐11*; and (3) a ‘double construct’ targeting *EpCH42:EpBAHD‐07:EpBAHD‐11* simultaneously. A construct silencing *EpCH42* independently was used as a control for each experiment.

**Fig. 4:**
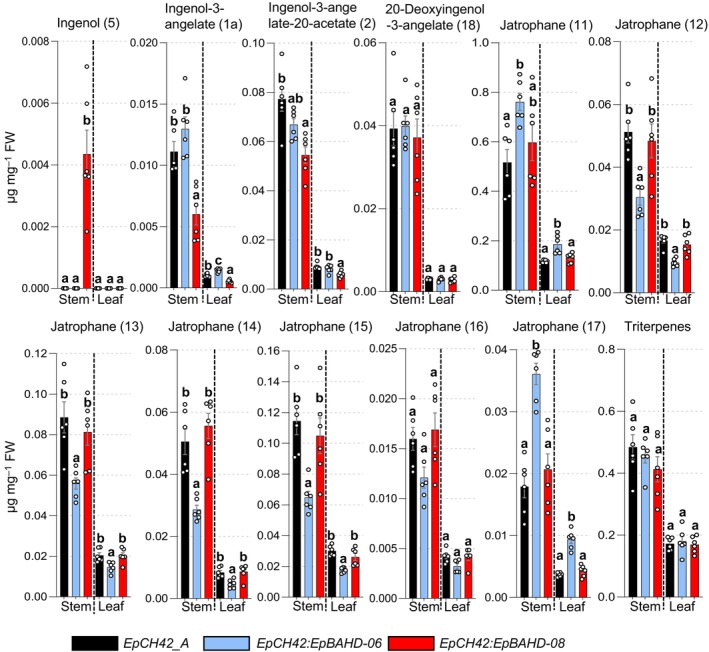
Virus‐induced gene silencing (VIGS) analysis of *EpBAHD‐06 and EpBAHD‐08* genes in *Euphorbia peplus*. Metabolite levels in VIGS material were measured for stem and leaves in VIGS marker‐only (*EpCH42_A*, black bars) and marker plus selected BAHD genes: *EpCH42:EpBAHD‐06* (cyan bars) and *EpCH42:EpBAHD‐08* (red bars). Triterpenes represent the sum of four major triterpenes annotated in Datasets S1 and S2. Error bars – SEM (*n* = 6). Letters represent Tukey's range test results after one‐way ANOVA was performed separately within the ‘leaf’ and ‘stem’ datasets. Groups not sharing letters within ‘leaf’ or ‘stem’ datasets indicate statistically significant differences (*P* < 0.05).

Chlorotic parts of stems and leaves were harvested *c*. 40 d post infiltration. Metabolite and transcript levels were then analyzed by LC‐MS and quantitative reverse transcription polymerase chain reaction (qRT‐PCR), respectively. Transcript levels of both *EpBAHD‐08* and *EpBAHD‐06* were significantly reduced (threefold) in stems compared with *EpCH42*‐infiltrated controls (Fig. S12A). However, expression of both BAHD genes was not significantly altered in leaves (Fig. S12B). Notably, all four *BAHDs* subjected to VIGS were expressed at low levels in leaves (10‐ to 20‐fold lower than stem), making detection of subtle changes in gene expression by qRT‐PCR unreliable (Fig. S12B). Next, we analyzed silencing effects on metabolite levels of ingenol‐3‐angelate (**1a**), ingenol‐3‐angelate‐20‐acetate (**2**), and ingenol (**5**), as well as a number of previously isolated ingenane and jatrophane diterpenoids (Metabolites **11**–**18**; Fig. S4) (Czechowski *et al*., 2022). Metabolite profiling clearly showed that silencing of *EpBAHD‐08* significantly decreased the levels of ingenol‐3‐angelate (**1a**) and ingenol‐3‐angelate‐20‐acetate (**2**) in leaves and stems of *E. peplus*, with the effect being more pronounced in stems in which diterpenoid concentrations are higher (Fig. 4; Dataset S1). Silencing of *EpBAHD‐08* has also led to the accumulation of ingenol (**5**) in stem material, a substrate for the *EpBAHD‐08* catalyzed reaction (Fig. 4; Dataset S1). VIGS thus corroborates the results obtained upon expression of *EpBAHD‐08* both *in vitro* and in *N. benthamiana* (Fig. 2b,d) and confirms the function of *EpBAHD‐08 in planta*, namely the angeloylation of ingenol (**5**) at the C3 position (Fig. 1b) to produce ingenol‐3‐angelate (**1a**). We thus named *EpBAHD‐08 ingenol‐3‐angelate synthase* (*I3AS*). Interestingly, the levels of 20‐deoxyingenol‐3‐angelate (**18**), another ingenane diterpenoid, remained unaltered by silencing of *EpBAHD‐08* (Fig. 4; Dataset S1), strongly suggesting that this compound is not directly derived from ingenol (**5**) and its biosynthesis does not involve *I3AS*.

By contrast, VIGS of *EpBAHD‐06* did not significantly alter the levels of any ingenanes in stems but had a clear effect on the level of jatrophane diterpenoids (Fig. 4). *EpBAHD‐06*‐silenced stems and leaves showed a significant increase of Jatrophanes 1 (**11**) and 7 (**17**), accompanied by a strong decrease of Jatrophanes 2–5 (**12–15**) content (Fig. 4; Dataset S1). It is noteworthy that the activity of *EpBAHD‐06* toward ingenol (**5**) observed in *N. benthamiana* and *in vitro* assays was very low when compared with the activity from *EpBAHD‐08* (Fig. 2b,d), implying it might represent a side activity for *EpBAHD‐06*. The VIGS results showing involvement of *EpBAHD‐06* in the biosynthesis of jatrophanes rather than ingenanes *in planta* is further confirmation of this.

Silencing of *EpBAHD‐07* and *EpBAHD‐11*, both of which showed activity on ingenol‐3‐angelate (**1a**), forming ingenol‐3‐angelate‐20‐acetate (**2**) in both *N. benthamiana* and *in vitro* assays, did not alter levels of any of the ingenanes in leaf or stem tissue (Fig. S13; Dataset S2). qRT‐PCR analysis showed only modest (1.5‐ to 1.8‐fold) transcript reduction in stem tissue, which was statistically significant for *EpBAHD‐11* transcript levels (Fig. S12C). Due to the redundant activity shown for *EpBAHD‐07* and *EpBAHD‐11*, a separate experiment was performed in an attempt to silence both genes simultaneously. Despite the stronger (twofold) and consistent, statistically significant reduction in *EpBAHD‐07* and *EpBAHD‐11* transcript levels in stem tissue (Fig. S12E,F), we did not observe any significant effect on level of ingenanes (Fig. S13; Dataset S3). Levels of some jatrophanes were slightly reduced in *EpBAHD‐11*‐silenced stem tissues (Fig. S13; Dataset S2), but this effect was not seen when *EpBAHD‐07* and *EpBAHD‐11* were silenced simultaneously (Fig. S13; Dataset S3), despite EpBAHD‐11 transcript levels being more strongly reduced in the latter experiment (Fig. S12E,F). Levels of triterpenes also remained unaltered in *EpBAHD‐07* and *EpBAHD‐11* VIGS experiments (Fig. S13; Datasets S2, S3).

As our VIGS results did not confirm that *Ep*BAHD‐07/*Ep*BAHD‐011 together are essential for the production of ingenol‐3‐angelate‐20‐acetate (**2**) *in planta*, we assumed there might be further genetic redundancy at this catalytic step in *E. peplus*. Upon closer inspection of our RNA‐seq data, we identified a close homologue of *Ep*BAHD‐07 (Fig. S14; hitherto referred to as *EpBAHD‐012*) that could potentially also be involved in the formation of **2**. Indeed, expression of *EpBAHD‐12* in *N. benthamiana* also led to acetylation of ingenol‐3‐angelate (**1a**) at C‐20, to produce ingenol‐3‐angelate‐20‐acetate (**2**) (Fig. S15). However, expression levels of *EpBAHD‐12* were unaltered in the *EpBAHD‐11* – silenced tissues, with its transcript levels being an order of magnitude lower than those of *EpBAHD‐07* (Fig. S12C–F). Lack of the *in planta* effect on ingenol‐3‐angelate‐20‐acetate (**2**) levels seen when *EpBAHD‐07* and *EpBAHD‐11* were silenced simultaneously could be explained by even greater functional redundancy in the *E. peplus* genome. In this regard, it is interesting to note that the *BAHD* gene family dramatically expanded during the evolution of land plants, with typically 50–200 BAHD copies present per genome (Moghe *et al*., 2023). This expansion has resulted in significant functional diversification, as witnessed by the presence of all seven subclades of BAHDs in the genomes of angiosperms (Moghe *et al*., 2023). It is therefore possible that additional functional homologues of *EpBAHD‐07*, *‐11*, and *‐12* are encoded in the *E. peplus* genome (Johnson *et al*., 2023). Such a high degree of redundancy would make VIGS challenging. Moreover, two‐ to threefold reductions in gene expression might not be sufficient to show a metabolite phenotype.

## Conclusion

Here, we report the discovery and characterization of the first genes linked to the committed steps of ingenol‐3‐angelate (**1a**) biosynthesis in *E. peplus*. We identified two genes, the products of which catalyze the addition of angelyl‐CoA (**9a**) to the ingenol (**5**) scaffold to produce ingenol‐3‐angelate (**1a**). We demonstrate using VIGS that just one of these genes, *Ep*BAHD‐08, is essential for this angeloylation in the native plant *E. peplus*. We also identified three genes whose products can catalyze the acetylation of ingenol‐3‐angelate (**1a**) to ingenol‐3‐angelate‐20‐acetate (**2**). In this case, VIGS indicates considerable functional redundancy in the *E. peplus* genome of genes encoding this enzymatic step. Silencing or knockout of these genes would enhance the production of ingenol‐3‐angelate (**1a**) *in planta* or give rise to nonacylated compounds, providing enhanced opportunity for semisynthesis of other biologically active ingenol‐type diterpenes. Notably, the steps leading to the formation of ingenol (**5**) from the putative intermediate jolkinol C (**7**) remain unclear. The discovery of the late steps of the ingenol biosynthetic pathway now provides a foundation for further discovery efforts in this pharmacologically important class of compounds.

## Competing interests

None declared.

## Author contributions

CS, PS, IAG and SOC designed the study. CS and MF performed all experiments, except those performed by RMA, TC and AG. RMA synthesized angelyl‐ and tiglyl‐CoA. TC designed, performed and analyzed the virus‐induced gene silencing experiments, helped by AG. YL performed phylogenetic analysis of the BAHD gene family. JW helped with molecular cloning. CS, TC, IAG and SOC wrote the manuscript. All authors read and agreed on the final version of this manuscript. CS and MF contributed equally to this work.

## Disclaimer

The New Phytologist Foundation remains neutral with regard to jurisdictional claims in maps and in any institutional affiliations.

## Supporting information


**Dataset S1** Levels of selected di‐ and triterpene metabolites in marker‐only (EpCH42) and marker plus EpBAHD‐06 and EpBAHD‐08 silenced (EpCH42:EpBAHD‐06 and EpCH42:EpBAHD‐08) stems and leaves.
**Dataset S2** Levels of selected di‐ and triterpene metabolites in marker‐only (EpCH42) and marker plus EpBAHD‐07 and EpBAHD‐11 silenced (EpCH42:EpBAHD‐07and EpCH42:EpBAHD‐11) stems and leaves.
**Dataset S3** Levels of selected di‐ and triterpene metabolites in marker‐only (EpCH42) and marker plus EpBAHD‐07 and EpBAHD‐11 simultaneously silenced (EpCH42:EpBAHD‐07:EpBAHD‐11) stems and leaves.

**Fig. S1** Chemical synthesis of ingenol‐3‐angelate from ingenol as reported by Liang *et al*. in 2012.
**Fig. S2** Structures of the major *Euphorbia* diterpene scaffolds jatrophane, lathyrane, tigliane, daphnane, and ingenane.
**Fig. S3** Biosynthesis of jolkinol C and jolkinol E in *Euphorbia peplus*.
**Fig. S4** Examples of (multi)acylated diterpenoids from *Euphorbia peplus*.
**Fig. S5** Observed levels of ingenol‐3‐angelate in different tissues of *Euphorbia peplus*.
**Fig. S6** Observed levels of ingenol‐3‐angelate‐20‐acetate in different tissues of *Euphorbia peplus*.
**Fig. S7** Fragmentation pattern analysis (MSMS) of ingenol‐3‐angelate and unknown product **1b** (peak a) using an authentic standard of **1a** for comparison.
**Fig. S8** Angelyl‐CoA ligase (*Ep*CCL2) is not required for ingenol‐3‐angelate formation in *Nicotiana benthamiana*.
**Fig. S9** Angelic acid is required for product formation in *Nicotiana benthamiana*.
**Fig. S10** Phylogenetic analysis of acyltransferases identified in this study.
**Fig. S11** Nucleotide alignment of 11 *BAHD*‐acyltransferase candidate genes candidate genes with virus‐induced gene silencing‐targeted regions highlighted in red for: *EpBAHD‐06*, *EpBAHD‐07*, *EpBAHD‐08*, and *EpBAHD‐11*.
**Fig. S12** Transcript abundance of selected genes in virus‐induced gene silencing experiments.
**Fig. S13** Virus‐induced gene silencing analysis of *EpBAHD‐07* and *EpBAHD‐11* in *Euphorbia peplus*.
**Fig. S14** Nucleotide and protein sequence alignment of *EpBAHD‐07* and *EpBAHD‐12*.
**Fig. S15** Transient expression of *Ep*BAHD‐12 in *Nicotiana benthamiana* in the presence of ingenol‐3‐angelate leads to formation of ingenol‐3‐angelate‐20‐acetate.
**Methods S1** Comprehensive descriptions of all materials and methods employed in the experiments in this study.
**Table S1** Co‐expression analysis of annotated BAHD‐acyltransferases from *Euphorbia peplus* with EpCAS.
**Table S2** Oligonucleotide primers used in this study for *Euphorbia peplus*.
**Table S3** Amino acid sequences of acyltransferase genes from *Euphorbia peplus* used in this study.
Please note: Wiley is not responsible for the content or functionality of any Supporting Information supplied by the authors. Any queries (other than missing material) should be directed to the *New Phytologist* Central Office.

## Data Availability

Gene sequence data have been deposited in GenBank (gene accession nos.: PQ801599–PQ801610). RNA‐seq data are available as GenBank BioProject PRJNA1214814. All other study data are included in the article and/or Figs S1–S15; Tables S1–S3; Datasets S1–S3.

## References

[nph70388-bib-0001] Alves ALV , Da Silva LS , Faleiros CA , Silva VAO , Reis RM . 2022. The role of ingenane diterpenes in cancer therapy: from bioactive secondary compounds to small molecules. Natural Product Communications 17: 1934578X2211056.

[nph70388-bib-0002] Brown D . 2016. Resiniferatoxin: the evolution of the “molecular scalpel” for chronic pain relief. Pharmaceuticals 9: 47. 27529257 10.3390/ph9030047 PMC5039500

[nph70388-bib-0003] Callari R , Fischer D , Heider H , Weber N . 2018. Biosynthesis of angelyl‐CoA in *Saccharomyces cerevisiae* . Microbial Cell Factories 17: 72. 29753326 10.1186/s12934-018-0925-8 PMC5948907

[nph70388-bib-0004] Czechowski T , Forestier E , Swamidatta SH , Gilday AD , Cording A , Larson TR , Harvey D , Li Y , He Z , King AJ *et al*. 2022. Gene discovery and virus‐induced gene silencing reveal branched pathways to major classes of bioactive diterpenoids in *Euphorbia peplus* . Proceedings of the National Academy of Sciences, USA 119: e2203890119. 10.1073/pnas.2203890119 PMC9173813 35584121

[nph70388-bib-0005] D'Auria JC . 2006. Acyltransferases in plants: a good time to be BAHD. Current Opinion in Plant Biology 9: 331–340. 16616872 10.1016/j.pbi.2006.03.016

[nph70388-bib-0006] Edgar RC . 2022. Muscle5: high‐accuracy alignment ensembles enable unbiased assessments of sequence homology and phylogeny. Nature Communications 13: 6968. 10.1038/s41467-022-34630-w PMC9664440 36379955

[nph70388-bib-0007] Hohmann J , Evanics F , Berta L , Bartók T . 2000. Diterpenoids from *Euphorbia peplus* . Planta Medica 66: 291–294. 10821064 10.1055/s-2000-8568

[nph70388-bib-0008] Jiang G , Mendes EA , Kaiser P , Wong DP , Tang Y , Cai I , Fenton A , Melcher GP , Hildreth JEK , Thompson GR *et al*. 2015. Synergistic reactivation of latent HIV expression by ingenol‐3‐angelate, PEP005, targeted NF‐kB signaling in combination with JQ1 induced p‐TEFb activation. PLoS Pathogens 11: e1005066. 26225771 10.1371/journal.ppat.1005066 PMC4520526

[nph70388-bib-0009] Johnson AR , Yue Y , Carey SB , Park SJ , Kruse LH , Bao A , Pasha A , Harkess A , Provart NJ , Moghe GD *et al*. 2023. Chromosome‐level genome assembly of *Euphorbia peplus*, a model system for plant latex, reveals that relative lack of Ty3 transposons contributed to its small genome size. Genome Biology and Evolution 15: 1–14. 10.1093/gbe/evad018 PMC10018070 36757383

[nph70388-bib-0010] Johnson HE , Banack SA , Cox PA . 2008. Variability in content of the anti‐AIDS drug candidate prostratin in samoan populations of *Homalanthus nutans* . Journal of Natural Products 71: 2041–2044. 19007283 10.1021/np800295m PMC2663895

[nph70388-bib-0011] Jørgensen L , McKerrall SJ , Kuttruff CA , Ungeheuer F , Felding J , Baran PS . 2013. 14‐Step synthesis of (+)‐ingenol from (+)‐3‐carene. Science 341: 878–882. 23907534 10.1126/science.1241606

[nph70388-bib-0012] King AJ , Brown GD , Gilday AD , Forestier E , Larson TR , Graham IA . 2016. A cytochrome P450‐mediated intramolecular carbon–carbon ring closure in the biosynthesis of multidrug‐resistance‐reversing lathyrane diterpenoids. Chembiochem 17: 1593–1597. 27272333 10.1002/cbic.201600316 PMC5095812

[nph70388-bib-0013] King AJ , Brown GD , Gilday AD , Larson TR , Graham IA . 2014. Production of bioactive diterpenoids in the Euphorbiaceae depends on evolutionarily conserved gene clusters. Plant Cell 26: 3286–3298. 25172144 10.1105/tpc.114.129668 PMC4371829

[nph70388-bib-0014] Kirby J , Nishimoto M , Park JG , Withers ST , Nowroozi F , Behrendt D , Rutledge EJ , Fortman JL , Johnson HE , Anderson JV *et al*. 2010. Cloning of casbene and neocembrene synthases from Euphorbiaceae plants and expression in *Saccharomyces cerevisiae* . Phytochemistry 71: 1466–1473. 20594566 10.1016/j.phytochem.2010.06.001

[nph70388-bib-0015] Liang X , Petersen AK , Högberg T . 2012. Semisynthesis of ingenol 3‐angelate (PEP005): efficient stereoconservative angeloylation of alcohols. Synlett 23: 2647–2652.

[nph70388-bib-0016] Luo D , Callari R , Hamberger B , Wubshet SG , Nielsen MT , Andersen‐Ranberg J , Hallström BM , Cozzi F , Heider H , Lindberg Møller B *et al*. 2016. Oxidation and cyclization of casbene in the biosynthesis of *Euphorbia* factors from mature seeds of *Euphorbia lathyris* L. Proceedings of the National Academy of Sciences, USA 113: E5082–E5089. 10.1073/pnas.1607504113 PMC5003294 27506796

[nph70388-bib-0017] Moghe G , Kruse LH , Petersen M , Scossa F , Fernie AR , Gaquerel E , D'Auria JC . 2023. BAHD Company: the ever‐expanding roles of the BAHD acyltransferase gene family in plants. Annual Review of Plant Biology 74: 165–194. 10.1146/annurev-arplant-062922-050122 36450296

[nph70388-bib-0019] Ronquist F , Teslenko M , van der Mark P , Ayres DL , Darling A , Höhna S , Larget B , Liu L , Suchard MA , Huelsenbeck JP . 2012. MrBayes 3.2: efficient Bayesian phylogenetic inference and model choice across a large model space. Systematic Biology 61: 539–542. 22357727 10.1093/sysbio/sys029 PMC3329765

[nph70388-bib-0020] Schotte C , Jiang Y , Grzech D , Dang TTT , Laforest LC , León F , Mottinelli M , Nadakuduti SS , McCurdy CR , O'Connor SE . 2023. Directed biosynthesis of mitragynine stereoisomers. Journal of the American Chemical Society 145: 4957–4963. 36883326 10.1021/jacs.2c13644 PMC9999412

[nph70388-bib-0021] Vasas A , Hohmann J . 2014. *Euphorbia* diterpenes: isolation, structure, biological activity, and synthesis (2008–2012). Chemical Reviews 114: 8579–8612. 25036812 10.1021/cr400541j

[nph70388-bib-0022] Vasilev VH , Spessert L , Yu K , Maimone TJ . 2022. Total synthesis of resiniferatoxin. Journal of the American Chemical Society 144: 16332–16337. 36043948 10.1021/jacs.2c08200

[nph70388-bib-0023] Wong J , de Rond T , d'Espaux L , van der Horst C , Dev I , Rios‐Solis L , Kirby J , Scheller H , Keasling J . 2018. High‐titer production of lathyrane diterpenoids from sugar by engineered *Saccharomyces cerevisiae* . Metabolic Engineering 45: 142–148. 29247866 10.1016/j.ymben.2017.12.007

[nph70388-bib-0024] Xu Y , Tang P , Zhu M , Wang Y , Sun D , Li H , Chen L . 2021. Diterpenoids from the genus Euphorbia: structure and biological activity (2013–2019). Phytochemistry 190: 112846. 34229224 10.1016/j.phytochem.2021.112846

[nph70388-bib-0025] Zhan Z , Li S , Chu W , Yin S . 2022. *Euphorbia* diterpenoids: isolation, structure, bioactivity, biosynthesis, and synthesis (2013–2021). Natural Product Reports 39: 2132–2174. 36111621 10.1039/d2np00047d

[nph70388-bib-0026] Zhao W , Wang F , Li P , Li L , Ernst L , Huang L , Tian M , Lv W , Xu S , Liu F *et al*. 2025. Two O‐acyltransferases from the diterpene biosynthetic gene cluster of *Euphorbia lathers* contribute to the structural diversity of medicinal macrocyclic diterpenoid esters biosynthesis. The Plant Journal 161: e70003. 10.1111/tpj.70003 39968625

